# Macrophages in Atheromatous Plaque Developmental Stages

**DOI:** 10.3389/fcvm.2022.865367

**Published:** 2022-04-25

**Authors:** Alexander von Ehr, Christoph Bode, Ingo Hilgendorf

**Affiliations:** ^1^Department of Cardiology and Angiology, University Heart Center Freiburg-Bad Krozingen, Faculty of Medicine, University of Freiburg, Freiburg, Germany; ^2^Institute of Experimental Cardiovascular Medicine, University Heart Center Freiburg-Bad Krozingen, Faculty of Medicine, University of Freiburg, Freiburg, Germany

**Keywords:** macrophage, monocyte, regression, trafficking, fate, atherosclerosis

## Abstract

Atherosclerosis is the main pathomechanism leading to cardiovascular diseases such as myocardial infarction or stroke. There is consensus that atherosclerosis is not only a metabolic disorder but rather a chronic inflammatory disease influenced by various immune cells of the innate and adaptive immune system. Macrophages constitute the largest population of inflammatory cells in atherosclerotic lesions. They play a critical role in all stages of atherogenesis. The heterogenous macrophage population can be subdivided on the basis of their origins into resident, yolk sac and fetal liver monocyte-derived macrophages and postnatal monocyte-derived, recruited macrophages. Recent transcriptomic analyses revealed that the major macrophage populations in atherosclerosis include resident, inflammatory and foamy macrophages, representing a more functional classification. The aim of this review is to provide an overview of the trafficking, fate, and functional aspects of the different macrophage populations in the “life cycle” of an atheromatous plaque. Understanding the chronic inflammatory state in atherosclerotic lesions is an important basis for developing new therapeutic approaches to abolish lesion growth and promote plaque regression in addition to general cholesterol lowering.

## Introduction

Atherosclerotic cardiovascular diseases such as myocardial infarction, stroke or peripheral artery disease remain the major cause of morbidity and mortality worldwide to date (1, 2). Despite scientific and therapeutic advance in recent years, there is still an endemic increase in cardiovascular diseases, especially in developing countries (1). Atherosclerotic lesions may occur in any arterial vessel, preferably at regions of non-laminar, low-shear flow (3), and progress slowly causing chronic symptoms such as stable angina or intermittent claudication. The rupture of a plaque or superficial erosions may lead to acute atherothrombotic vascular occlusions with life-threatening consequences such as myocardial infarction or stroke. The standard therapeutic pillars of atherosclerosis primary and secondary prevention therefore encompass antithrombotic and cholesterol lowering treatment options. Yet, despite ever lower target levels for low density lipoprotein cholesterol, residual cardiovascular risk remains prevalent. Vascular inflammation is considered to contribute to the persistent risk of recurrent atherothrombotic events. Macrophages are critically involved in the formation and progress of atherosclerotic lesions. Although some argue that without cholesterol there is no atherogenesis, experimental studies with C-C chemokine receptor 2 (CCR2) deficient mice indicate that lack of lesion infiltrating monocytes, which give rise to plaque macrophages, protects against atherosclerosis even in the presence of hypercholesterolemia (4, 5). Monocytes are also attracted to atherosclerotic lesions *via* chemokine receptors CCR5 and CX3CR1 (4, 6, 7). Overall restriction of leukocyte infiltration by disruption of CD40 and CD40L interaction and other adhesion molecules attenuates atherosclerosis formation (8–10). According to the current pathomechanistic concept, endothelial dysfunction and activation alongside accumulation of oxidatively modified lipoproteins in the subintimal space of arterial vessels facilitate inflammatory cell recruited to the nascent atheromatous plaque. Beyond new cell recruitment, macrophages and transdifferentiated vascular smooth muscle cells proliferate locally, form foam, cells and propagate disease progression (11–13). In this work we review the roles of arterial macrophage subtypes during the course of atherogenesis highlighting novel insights from fate mapping studies.

## Macrophage Origins in the Vasculature

**FIGURE 1:**
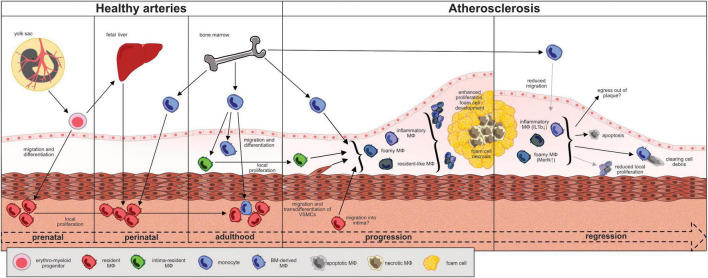
Fate, trafficking and functional changes of macrophages during the “life cycle” of an atheromatous (murine) plaque. EMP-derived macrophages seed the tissues between day E9 and E12 of embryonic development. Macrophages reside primarily in the adventitia and sustain through local proliferation. Monocyte-derived macrophages from the fetal liver and, around birth, from the bone marrow seed the vessel wall and differentiate in the adventitia. The recently introduced intima resident macrophages (Mac^AIR^) originate from the bone marrow and maintain through local proliferation. They are the first macrophages to get in contact with lipoproteins in the intima and are outnumbered and replaced by newly recruited monocyte-derived macrophages during atherogenesis. At advanced disease stages, local macrophage proliferation dominates cell turnover. Vascular smooth muscle cells may transdifferentiate and adapt a macrophage-like phenotype and contribute to the atheromatous foam cell population. The role of adventitial macrophages in this context remains to be elucidated. Major atheromatous plaque macrophage subpopulations, as defined by their transcriptomes, include resident (*Ccl8, Lyve-1, FOLR2*, CXCL4, *F13a1, Wfdc17*), inflammatory (*Cxcl2, Ccl2, Ccl3, Ccl4, Il-1b, TNF*), and TREM-2^+^ foamy (*Lgals3, Cd9, Ctsd*) macrophages. The degree of plasticity in between subpopulations and their respective roles in disease progression are under investigation. In murine models of atherosclerotic regression, inflammatory macrophages showed less Nlrp3 and IL1β expression while Trem2^hi^ foamy macrophages expressed more Mertk and less Mmp2 in line with a rather atheroprotective function.

For decades macrophages, first described in the late nineteenth century, were believed to arise exclusively from monocytes infiltrating tissues and giving rise to tissue macrophages. While monocyte differentiation into macrophages appears to be particularly relevant in the context of tissue inflammation, recent fate mapping, proteomic, and transcriptomic single cell profiling studies in mice portray a far more divers story on macrophage origins and heterogeneity. Broadly speaking, both embryonic, yolk sac (YS)- and fetal liver monocyte-derived resident macrophages, and postnatal monocyte-derived recruited macrophages contribute to varying degrees to the pool of macrophages in different tissues (14–18). Macrophages are the first innate immune cells to seed the tissues between day E9 and E12 of embryonic development in mice, including the arterial vascular system (19–22). These prenatally derived, resident macrophages are long-lived and their population self-sustains by local proliferation predominantly in the adventitia in adulthood (Figure 1) (21). Other macrophage populations in arteries arise after birth from infiltrating monocytes that originate from bone marrow (BM) hematopoiesis and populate both the intima and adventitia (21, 23). Monocyte-derived macrophages become even more prevalent during atheromatous plaque development, phagocytizing oxidized lipoproteins and apoptotic cells inside the plaque, and thereby critically regulating local inflammation. These leukocyte-derived macrophages in the plaque are complemented by macrophage-like cells that originate from infiltrating vascular smooth muscle cells (SMC) through transdifferentiation, adopting some macrophage signature markers and forming foam cells (Figure 1) (24). Much of our current knowledge of macrophage ontogeny is based on fate mapping studies in animal models such as mice or zebrafish. Because similar studies in early human embryos are limited, embryonic development and characterization of the various macrophage subtypes in humans remain poorly understood. Phenotypic analyses showed that macrophages in the human YS at week 9 of gestation appear as spherical as murine macrophages and acquire a typical phagocytic phenotype with multiple dendrites during maturation. Furthermore they express similar macrophage markers (CD68, CX3CR1) as their counterparts in mice (20). Recent single-cell RNA sequencing from early human embryos obtained after abortion at different time points of development revealed two waves of YS-derived embryonic tissue resident macrophages. The first wave of primitive yolk sac-derived macrophages were detected in the yolk sac at Carnegie stage 11 (CS11), whereas yolk sac derived myeloid progenitor cells give rise to a second monocyte-derived macrophage population after CS17 (25). These findings resemble data acquired in murine studies, suggesting a relevant concordance in macrophage development between mice and human. In the following we discuss how macrophages of diverse origins contribute to vascular health and disease.

## Macrophages in Healthy Arteries

The arterial vessel wall is composed of three layers: the innermost intima containing the endothelium as a barrier to the blood stream, the media predominantly consisting of vascular smooth muscle cells (VSMCs) and the outer and mostly fibrous adventitia. Under physiological conditions, the vast majority of arterial wall macrophages reside in the adventitia. The intima, the site where atheromatous plaques develop, harbors less than 10% of the overall arterial macrophage population under healthy conditions (19, 31). In mice the YS gives rise to erythro-myeloid progenitors (EMP) which colonize the cardiovascular system and develop into tissue resident macrophages (Figure 1) (21, 32). Around birth, almost all macrophages in the arterial wall derive from YS EMP and fetal liver monocytes (21). Postnatally, bone marrow-derived blood monocytes invade the arterial wall and contribute to the macrophage population both in the adventitia and intima (21, 23). Interestingly, the dual origin of adventitial macrophages is maintained by local proliferation and cell recruitment at a ratio of about 70% prenatally derived and 30% monocyte-derived macrophages which slowly declines with age (19). Recent scRNA-sequencing analyses of murine aortic cell suspensions identified different subsets among CD45^+^, Csf1r^+^, MerTK^+^, and CD64^+^ macrophages in the healthy aorta. Resident macrophages in the adventitia typically express high levels of Lymphatic vessel hyaluronan receptor-1 (Lyve-1) with some subsets presenting with an interferon-inducible signature (Isg15+, Stat1+) or specialized in antigen presentation. The role of resident adventitial macrophages for maintaining vascular health has yet to be fully elucidated, but scRNA-Seq-analyses point toward homeostatic functions based on high expression levels of Lyve-1, Growth arrest-specific protein 6 (Gas6) and Stabilin-1 (Stab1). Csf1r blockade in mice leads to aortic macrophage depletion and results in increased arterial stiffness and fibrosis, effects reversed upon repopulation with arterial macrophages (33). Adventitial macrophage-derived collagenolytic MMP9 release depends on Lyve-1-hyaluronan interactions with smooth muscle cells, and Lyve-1 expressing macrophages can promote angiogenesis in mice and human (33, 34). Macrophage-expressed Gas6 and Stab1 act as scavenger receptors mediating the clearance of apoptotic cells (35, 36). A small subset of less than 10% of murine aortic macrophages features a distinct profile enriched for inflammatory markers such as interleukin-1β (IL-1b) and MMP12. It develops from monocytes, largely resides in the intima and was therefore termed “aorta intima resident macrophages” (Mac^AIR^) (23). Mac^AIR^ are predominantly found in arterial branches, locations of turbulent flow, and more susceptible to atherosclerosis development (23). Monocytes, classified as Ly6C^high^ CCR_2_^+^ and Ly6C^low^ CCR_2_^–^ subsets in mice, play a subordinate role for aortic macrophage maintenance during homeostasis. Merely 20% of the arterial macrophage pool is constantly replenished by invading blood monocytes, predominantly by the Ly6C^high^ subset (19, 31). Cycling Ly6C^low^ monocytes constantly surveil the endothelium but rarely cross the endothelial barrier (37–39). Due to the pro-inflammatory properties of monocyte-derived macrophages it is conceivable that BM-derived macrophages represent the vascular first line of defense against invading pathogens whereas EMP-derived macrophages exert primarily homeostatic functions, as described above. In keeping with this concept, the population of resident adventitial macrophages outlasts acute inflammatory challenges (19, 21). A single injection of LPS or continuous infusion of Angiotensin II (Ang II) cause acute vascular inflammation in mice and a transitory surge in monocyte-derived macrophages in the arterial wall. However, within days to weeks the original, resident macrophage composition of predominantly embryonically-derived macrophages is being restored through enhanced local proliferation (19, 21). Functionally, EMP-derived macrophages in the adventitia express genes associated with tissue repair during Ang II infusion whereas monocyte-derived macrophages activated inflammatory processes (19).

These findings are in line with previous murine studies from the heart. Macrophages populate the heart already during embryonic development, self-sustain through *in situ* proliferation and orchestrate cardiac tissue homeostasis (40). In an acute cardiac injury, the resident cardiac macrophages of prenatal origin support healing after myocardial infarction, whereas macrophages that descend from recruited monocytes aggravate adverse cardiac remodeling (41). In general, macrophage ontogeny defines distinct cellular programs in- and outside the vessel (18, 42).

Hu et al. recently analyzed the transcriptomic profile of macrophages in different healthy human cardiac arteries after heart transplantation (29). Four macrophage subsets were identified (inflammatory macrophage 1, inflammatory macrophage 2, resident macrophage, and dividing macrophage). Inflammatory macrophages mainly expressed typical M1 markers, such as TNF, IL1B, CCL3, and CXCL8 whereas M2 markers were scarcely expressed in any of the macrophage subsets (29). The authors concluded that even under physiological conditions a majority of macrophages in human cardiac arteries feature an inflammatory profile with activation of NFKB pathways and inflammasome regulation (29, 43).

## Macrophages and the Progression of Atherosclerosis

Atherosclerosis is a major pathomechanism underlying cardiovascular disease, including coronary and ischemic heart disease, peripheral artery disease, and stroke. Oxidized low density lipoproteins accumulate in the arterial intima sparking a chronic, lipid-driven inflammatory response, enhanced by shear stress, and hypoxia (44–48). In response, monocytes infiltrate the intima, differentiate into atheromatous plaque macrophages, proliferate locally, and phagocytize lipoproteins leading to foam cell formation (27, 49, 50). Hypercholesterolemia, inflammation, sedentary life style, and mental stress stimulate medullary and extramedullary hematopoiesis resulting in higher numbers of circulating pro-inflammatory monocytes (51–56). Ly6C^low^ monocytes appear to be better equipped for lipid uptake in the circulation which enhances their patrolling behavior and aids in atheroprotection (37, 57), while Ly6C^high^ monocytes are more prone to infiltrate the arterial vessel wall (11). The recruitment of monocytes from the bloodstream to the intima is mediated by chemokine receptors (CCRs), including CCR2, CCR5, and CX3CR1 in mice and human (7, 58, 59). Endothelial, smooth muscle, and immune cells as well as platelets secret chemokine ligands of the CC family (CCLs) that interact with the respective chemokine receptor on the monocyte surface (58, 60). Ly6C^high^ monocytes express low levels of CX3CR1 and high levels of CCR2 (61), but secretion of CCL2 by endothelial cells can enhance monocyte CX3CR1 expression and facilitate their adhesion *via* integrins (62–64). After crossing the endothelial barrier, Ly6C^high^ monocytes, beyond differentiating into tissue macrophages, may proliferate *in situ* or function as antigen-presenting cells themselves when migrating to lymph nodes (65, 66). Murine Ly6C^high^ monocytes resemble human CD14^++^/CD16^–^ monocyte population, whereas Ly6C^low^ monocytes correspond to the CD14^+^/CD16^++^ subset (60).

Recently described intima resident Mac*^AIR^* become foam cells even before blood monocytes are recruited to the nascent plaque. Transgenic mice lacking Mac*^AIR^* show less lipid deposition in the first stages of atherosclerosis suggesting a pioneering role at the onset of atherogenesis (31). As atherosclerosis progresses, Mac*^AIR^* are marginalized by atheromatous plaque macrophages derived from newly recruited monocytes and transdifferentiating vascular smooth muscle cells (VSMC) (11, 31). In advanced plaques of Apoe^–/–^ mice, all macrophages of monocyte origin renew within 4 weeks, but largely independent of newly recruited monocytes (13, 50). Instead, more than 85% of atheromatous plaque macrophages renew through local proliferation (13). Non-leukocytes expressing prototypical macrophage markers and forming foam cells may originate from VSMCs. Under physiological conditions, they express typical SMC markers such as α-smooth muscle actin (α-SMA). During atherogenesis in human carotid arteries VSMCs exhibit a loss in contractility, a higher proliferation rate and a reduced expression of α-SMA (67). VSMCs constitute at least 30% of all plaque cells (68) and their contribution may be even underestimated as they switch their phenotype. Lineage-tracing approaches in mice indicate that VSMC are able to adopt characteristics of osteoblast, fibroblasts, and macrophages (69). VSMC play a major role in cell-cell-interactions attracting various immune cells to the plaque by the secretion of cytokines such as IL-6, CCL2, and upregulation of ICAM-1 and fostering chronic inflammation of the arterial vessel wall (70). Equipped with scavenger receptors for lipoprotein uptake, VSMC account for the majority of foam cells in the plaque, although only a fraction of these cells co-expresses macrophage markers which classify them as macrophage-like cells (71). VSMC-specific deletion of the transcription factor Krüppel-like factor 4 (KLF4) limits transdifferentiation of VSMCs into macrophage-like cells and slows plaque progression (72). It is estimated that about 40% of cells expressing macrophage marker CD68 in human plaque derive from VSMC, but the majority of atheromatous plaque macrophages arises from monocytes and *via* local proliferation (73).

The traditional view was Ly6C^high^ monocytes differentiate into macrophages with M1 type features during the progression of atherosclerosis, releasing pro-inflammatory cytokines (TNF-α, IL-12, IL-6) and reactive oxygen species, while differentiation into alternatively activated M2 type macrophages facilitates plaque regression (74). Recent single cell analyses of atheromatous plaque immune cells using mass cytometry and RNA sequencing refuted the dichotomous M1/M2 classification and instead identified macrophage subsets with mixed phenotypes specialized in inflammation, lipid handling, and homeostasis (26, 28, 30). For example, in mice the expression of transcription factor Interferon regulatory factor 5 (IRF 5), mediating classical M1 type macrophage polarization *in vitro*, is not restricted to the inflammatory macrophage subpopulation in the atherosclerotic aorta (30). Loss of Irf5 in myeloid cells limits lipid and macrophage accumulation in the plaque, decreases IL-12 and increases TGFβ, MerTK, and CD206 expression, promoting a stable plaque phenotype (30, 75, 76). Major macrophage populations in murine atherosclerotic aortas, as defined by their transcriptomes, include resident (*Ccl8, Lyve-1, FOLR2* CXCL4,*F13a1, Wfdc17*), inflammatory (*Cxcl2, Ccl2, Ccl3, Ccl4, Il-1b, TNF*), and TREM-2^+^ foamy (*Lgals3, Cd9, Ctsd*) macrophages (Table 1), and smaller populations such as interferon-inducible (*Ccl12, Isg15, Irf7, Ifit1, Ifit3*) and cavity macrophages (*Fn1, Clec4b1, Sept11, Ear2*) (26, 27). The latter shares transcriptional similarities with macrophages from the peritoneum and may actually infiltrate from pleural or pericardial cavities. Their role and impact in atherosclerosis remains elusive to date. Transcriptionally, proliferating atheromatous plaque macrophages represent a distinct subpopulation without preferential overlap with one of the aforementioned major macrophage subsets in mice and men (77, 78). These data do not indicate whether one macrophage subset is more prone to proliferate than others or what may trigger local proliferation, which dominates cell turnover in advanced atherosclerotic lesions. However, we have identified scavenger receptors Msr1 and CD36, involved in the uptake of modified lipoproteins, experimentally as mediators of local macrophage proliferation in atherosclerotic aortas using a mixed bone-marrow chimeric approach in mice (12). In line, *in situ* macrophage proliferation in human carotid artery plaques correlated with serum LDL-cholesterol levels and plaque lipid contents (12). In addition, macrophage colony-stimulating factor or uptake of apoptotic cells stimulate proliferation of plaque macrophages and the latter induces an inflammation-resolving phenotype (79–81). CD47 binding to inhibitory signal regulatory protein α (SIRPα) on macrophages induces a “don’t eat me” signal. SIRPα deletion on macrophages enhances their efferocytic capacity and suppresses atherogenesis in mice similar to effects observed with the administration of CD47-blocking antibodies (82, 83).

**TABLE 1:** scRNA-seq from murine and human arteries identified markers that classify macrophage subsets into inflammatory, resident-like, and foamy macrophages.

	Murine marker	Human marker	Proposed function
Inflammatory macrophages	Tnf, Ccl2, Ccl3, Ccl4, Cxcl2 (26, 27)	TNF, IL1b, CASP1, CASP4, CCL3, CCL4 (28, 29)	Pro-inflammatory
Resident-like macrophages	Lyve1, Cx3cr1^hi^, Cd206, Gas6, Ccl8, CXCL4, Wfdc17 (26, 27)	TXNIP, Cd14, YWHAH (29)	Regulatory, endocytosis
Foamy macrophages	Trem2, Cd9, Lgals3, Fabp5, Ctsd, Abcg1 (26, 30)	ABCA1, ABCG1, MMP9, OLR1 (28, 30)	Both pro- and anti-inflammatory, cholesterol metabolism

*The proposed functions of different subsets are yet to be confirmed experimentally.*

Although foam cells are considered a hallmark of atherosclerotic plaques, and high plaque lipid loads represent a feature of plaque vulnerability, TREM-2 + foam cells feature a rather anti-inflammatory profile and enhanced lipid metabolism in contrast to inflammatory macrophage populations (84). TREM-2 is a transmembrane receptor that interacts with apolipoprotein E and its expression is enhanced by alternative macrophage activation *via* the IL-4 pathway and abolished by LPS and IFN-γ as pro-inflammatory cytokines (85, 86). Experimental models have shown that cholesterol mediated foam cell formation deviates the cellular cholesterol-biosynthetic pathway resulting in the preferential production of desmosterol, which suppresses inflammatory responses in macrophages (87). Conversely, overexpression of the enzyme converting desmosterol into cholesterol in an atherosclerosis mouse model stimulated inflammasome activation in plaque macrophages while even enhancing intracellular lipid accumulation, thus decoupling foam cell formation from inhibition of inflammation (88). Other means of foam cell formation, e.g., oxidized LDL uptake *via* CD36, have also demonstrated to trigger rather than suppress inflammation (89). Activation of the olfactory receptor 2 on vascular macrophages by octanal, a lipid aldehyde formed during oxidative stress and lipid accumulation in atherosclerotic plaques, stimulates the inflammasome and exacerbates atherosclerosis in mice (90). Finally, a more refined algorithm analyzing human atherosclerotic tissue single cell transcriptomic datasets was able to discriminate two distinct programs in plaque macrophages—homeostatic foaming and inflammatory pathogenic foaming. Gene expression profiles associated with inflammatory foaming correlate with cardiovascular events in the Multi-Ethic Study of Atherosclerosis, indicating that foam cell formation and inflammation are not mutually exclusive after all (91). The degree of cell plasticity which allows macrophages to adopt different phenotypes within atherosclerotic plaques remains an active field of investigation. Likewise, it is unclear to what extend the prominent population of resident-like Lyve-1 + macrophages, identified by unsupervised scRNA-Seq of atherosclerotic tissues, derives from adventitial macrophage precursors of largely prenatal origin or from recruited monocytes. A large fraction of resident-like macrophages expresses CLEC4A2, a C-type lectin receptor. Depletion of CLEC4A2-expressing macrophages, located mainly in the adventitia, increased atherosclerotic lesion formation in mice, pointing toward an atheroprotective role by preserving lipid handling and suppressing inflammatory responses (92).

In advanced atherosclerotic lesions, male ApoE^–/–^ mice have greater numbers of macrophages than their female littermates. However, at week 22 of a proatherogenic diet, the number of macrophages in female ApoE^–/–^ mice is similar to or higher than in males. Elizabeth Moss et al. recently showed that female Ldlr-deficient mice in an AAV-PCSK9 mouse model develop larger atherosclerotic plaques but are protected from vascular inflammation. Compared with males, they have 62% fewer myeloid cells in the aortic arch (93). The finding of lower plaque inflammation in female mice is consistent with a recent study that found lower expression of matrix metalloproteinase-12 (MMP12) by lesional macrophages in aortic root plaques from female mice compared with males (94). The lower plaque inflammation in female mice may indicate a more stable plaque phenotype and explain the protection from cardiovascular ischemia observed in premenopausal women (95). Various studies of human carotid endarterectomy specimen showed that atherosclerotic plaques from men contain more macrophages than women of the same age, regardless of disease severity or symptomatology (96–98). Although plaque macrophage numbers may differ between males and females, sex-dependent differences in transcriptional profiles require further studies and may be confounded by the extent of disease. Of note, in the heart, little to no difference between male and female human macrophages were detected apart from genes on X and Y chromosomes (99).

## Macrophages and the Regression of Atherosclerosis

While we have not harbored the full potential of primary prevention, yet, many patients present to the doctor already with established atherosclerotic cardiovascular disease. Therefore, promoting regression of atherosclerotic lesions is of utmost clinical importance. In contrast, mechanisms that govern plaque regression are less well studied than those driving plaque progression. Clinical trials using intravascular imaging tools have shown that plaque volumes decline minimally but plaque composition can change significantly upon reduction of LDL-cholesterol levels below 70 mg/dL (1.8 mmol/L), with fibrotic cap thickness increasing and lipid and macrophage contents decreasing (100, 101). Reversal of elevated cholesterol levels in atherosclerotic mouse models was used to study plaque regression, experimentally, by switching from high-cholesterol to cholesterol-free diets, transplanting atherosclerotic aortas into non-hypercholesterolemic mice or interfering with hyperlipidemia associated genes on a transcriptional level (102–104). In humans, however, significant and lasting reductions in LDL cholesterol levels depend on drug treatment which target cholesterol biosynthesis (e.g., statins, bempedoic acid), absorption (ezetimibe) or hepatic LDL particle clearance (PCSK9 inhibition) (105). The ApoE3L.CETP transgenic mouse model features a humanized lipid profile and responds to oral statin treatment with plasma cholesterol lowering unlike the more common atherosclerosis mouse models with ApoE and Ldlr-deficiency. In ApoE3L.CETP mice with established atherosclerosis, oral atorvastatin treatment induced plaque regression with marked decline in macrophage content. Although monocyte infiltration was reduced, as previously reported (23, 106), the reduction in atheromatous plaque macrophage numbers mostly depended on the suppression of local proliferation and constant cell death (12). Macrophage egress from sites of inflammation (107), an alternative mechanism to explain the decline in macrophage numbers during plaque regression, was observed in some (74, 108, 109), but not other studies (12, 23, 106, 110). Aside from numeric changes, transcriptional profiles of murine macrophages in regressing plaques were compared to those in progressing plaques. Interestingly, proportions of resident-like, inflammatory and foamy macrophages, as defined by bioinformatic cluster and marker gene analysis, were relatively similar in progressing and regressing plaques. Macrophage genes associated with plaque progression encoded for MHCII molecules and Malat1, while Cathepsins and Cxcr4 were overexpressed during regression (65). Inflammatory macrophages expressed less Nlrp3 and IL1b while foamy macrophages expressed more Mertk and less Mmp2 (111). Smaller subpopulations with interferon- and IL-4-signatures were more prevalent in progressing plaques, indicative of more diversified cell polarization states during plaque progression and revising a previous oversimplified concept of M1–M2 macrophage conversion during plaque regression (65, 74). Still, administration of M2-polarizing IL-13 can induce plaque regression or slow progression in mice with decreased plaque macrophage numbers and improved cellular lipid handling independent of systemic cholesterol levels (110). More physiologically, regulatory T cells accumulate in regressing plaques and support macrophage egress and apoptosis while suppressing macrophage proliferation. Tregs enhanced the efferocytic and inflammation-resolving capacity of plaque macrophages, and their depletion prevented plaque regression in mice even when serum cholesterol levels were normalized (111). Under hyperlipidemic conditions, however, plaques continue to progress as protective Tregs covert into pathogenic T_*H*_1/T_*H*_17 cells (112). These data encourage the design and study of atherosclerosis vaccines to support atherosclerosis regression (112, 113).

## Therapeutic Approaches Targeting Macrophages

As described above, macrophages play a crucial role in all phases of the atheromatous plaque’s “life cycle,” and thus represent potential therapeutic targets. In atherosclerotic mice, insufficient cholesterol efflux from cells was linked to enhanced hematopoietic activity and an increase in hematopoietic stem and progenitor cells (HSPC) and their progenies (52, 114). Systemic monocytosis leads to increased recruitment of macrophages to the vessel wall and the number of macrophages in the plaque correlates with plaque vulnerability: In general, the more macrophages in the plaque, the more susceptible it is to rupture (12, 115). Systemic therapy with anti-IL1b antibodies decreases monocyte and neutrophil blood counts in experimental models and patients post myocardial infarction, and suppresses endothelial cell activation (54, 116). Of note, in the CANTOS trial treatment with the anti-IL1b-antibody canakinumab significantly reduced rates of recurrent cardiovascular disease, independent of alterations in lipid levels (117).

Upstream of IL1b release by macrophages, the inflammasome, a macromolecular structure composed of multiple components responsive to intra- or extracellular danger signals (118), activates caspase1, which cleaves the pro-form of IL-1b. In particular, cholesterol crystals, hypoxia, and non-laminar flow can lead to inflammasome and caspase1 activation in the plaque; usually several of these stimuli are required for sustained activation (119–122). Expression of Nlrp3 inflammasome components in atherosclerotic plaques of patients correlates with cardiovascular disease severity (123), and NLRP3 expression in blood monocytes predicts adverse cardiac events (124). Several small molecular inhibitor trials targeting the NLPR3 inflammasome are currently underway. Current guidelines recommend considering low dose colchicine treatment for secondary prevention in patients at very high cardiovascular risk following a series of positive clinical trials (125–127). Although the exact mechanisms underlying colchicine’s atheroprotective effects are not well understood, inhibition of the NLRP3 inflammasome appears to be a plausible mode of action.

We propose that inhibition of local macrophage proliferation in atherosclerotic lesions may support plaque regression. This may be achieved by reducing cholesterol-rich lipoprotein levels which we recently identified as triggers of macrophage proliferation (12) or, potentially, by promoting plaque cholesterol efflux with recombinant Apolipoprotein A infusions, an approach being tested in the AEGIS II cardiovascular outcome trial. Alternatively, nanoparticles can be used to deploy antiproliferative or gene expression modifying agents in atherosclerotic lesions to target plaque macrophages directly (128, 129) and to prevent or even revert disease progression.

## Concluding Remarks

Macrophages are prominent cells in the pathogenesis of atherosclerosis. Thanks to their versatile nature, they carry both inflammation propagating and resolving properties. Decreasing triggers of plaque progression, e.g., dyslipidemia, while fostering reparative cell responses through immunomodulation and targeted nanoparticle approaches may help to promote plaque regression in the future (112, 113, 130, 131).

## Author Contributions

AE and IH contributed equally to writing the manuscript. CB discussed and commented the research. All authors contributed to the article and approved the submitted version.

## Conflict of Interest

The authors declare that the research was conducted in the absence of any commercial or financial relationships that could be construed as a potential conflict of interest.

## Publisher’s Note

All claims expressed in this article are solely those of the authors and do not necessarily represent those of their affiliated organizations, or those of the publisher, the editors and the reviewers. Any product that may be evaluated in this article, or claim that may be made by its manufacturer, is not guaranteed or endorsed by the publisher.
